# Revisiting the Hygiene Hypothesis in the Context of Autoimmunity

**DOI:** 10.3389/fimmu.2020.615192

**Published:** 2021-01-28

**Authors:** Jean-François Bach

**Affiliations:** ^1^Université de Paris, Paris, France; ^2^INSERM U1151, Institut Necker-Enfants Malades, Paris, France; ^3^Academie des Sciences, Paris, France

**Keywords:** hygiene hypothesis, autoimmune diseases, type 1 diabetes, non-obese diabetic mouse, Toll-Like Receptor, gut microbiota, evolution, migrants

## Abstract

Initially described for allergic diseases, the hygiene hypothesis was extended to autoimmune diseases in the early 2000s. A historical overview allows appreciation of the development of this concept over the last two decades and its discussion in the context of evolution. While the epidemiological data are convergent, with a few exceptions, the underlying mechanisms are multiple and complex. A major question is to determine what is the respective role of pathogens, bacteria, viruses, and parasites, *versus* commensals. The role of the intestinal microbiota has elicited much interest, but is it a cause or a consequence of autoimmune-mediated inflammation? Our hypothesis is that both pathogens and commensals intervene. Another question is to dissect what are the underlying cellular and molecular mechanisms. The role of immunoregulatory cytokines, in particular interleukin-10 and TGF beta is probably essential. An important place should also be given to ligands of innate immunity receptors present in bacteria, viruses or parasites acting independently of their immunogenicity. The role of Toll-Like Receptor (TLR) ligands is well documented including *via* TLR ligand desensitization.

## Introduction

The hygiene hypothesis is a counterintuitive concept. While it is well known that infectious agents are potentially responsible for many diseases beyond infectious diseases, the idea emerged that they could in some cases have a favorable effect on non-infectious and sometimes very serious illnesses. The original report by Strachan in 1989 was based on an observation that might seem anecdotal: hay fever and atopic dermatitis are less frequent in families with many children than in families with only one or two children (1). It was to Strachan’s credit that he then proposed the hypothesis that common childhood infections may reduce the frequency of atopic diseases. It was only a little later, in 2000, that he proposed that the increase in the frequency of allergic diseases observed in the three or four preceding decades could be ascribed to the decrease in the frequency of infectious diseases (2). It was also in the early 2000s that the hygiene hypothesis was extended to autoimmune diseases (3). At that time there were already data obtained in experimental models showing that infections, particularly parasitic infections, could prevent the occurrence of autoimmunity (4). Since then, compelling evidence has been gathered to support the hygiene hypothesis. We review it here stressing the importance of causal relationships, since it is not sufficient to show a correlation between two events to affirm causality. We will highlight the importance of experimental models, particularly those concerning spontaneous diseases, the closest to human diseases.

Like any scientific hypothesis, the hygiene hypothesis has elicited conflicting opinions. By examining several hundred articles devoted to the subject, we find a majority supporting the hypothesis. There are, however, a number of articles expressing reservations or even, more directly, questioning the hypothesis. These challenging reports relate particularly to allergic diseases. Many allergists are more inclined to explain the increase in the frequency of allergic diseases, which nobody denies, by changes in the non-infectious environment, even going so far as to incriminate the increase in the dissemination of pollens. Such claims are difficult to accept when one considers that the increase in the frequency of allergic diseases affects all clinical forms ranging from atopic dermatitis to hay fever and even food allergies. In addition, the parallel evolution of autoimmune diseases does not support the hypothesis of changes in the allergenic environment. Another source of questioning is linked to the fact that all autoimmune diseases are not concerned by the hypothesis, without knowing why some of them are and others not, a subject of very great interest *per se*. Also, it is very difficult to know which infections are involved in the hypothesis. The study of experimental models makes it possible to identify infectious agents, bacteria, viruses and especially parasites, which prevent the occurrence of allergic and autoimmune diseases. Analysis is much more difficult in humans. The lower incidence of allergic and autoimmune diseases in large families mentioned above suggests that common childhood infections play a role. The mirroring chronological course of the decrease in major infectious diseases and the increase in allergic and autoimmune diseases argues for serious infectious diseases being also involved in the hypothesis. The problem is further complicated by the fact that certain infectious agents can cause acute autoimmune diseases as rheumatic fever and Guillain–Barré syndrome.

**Figure 1:**
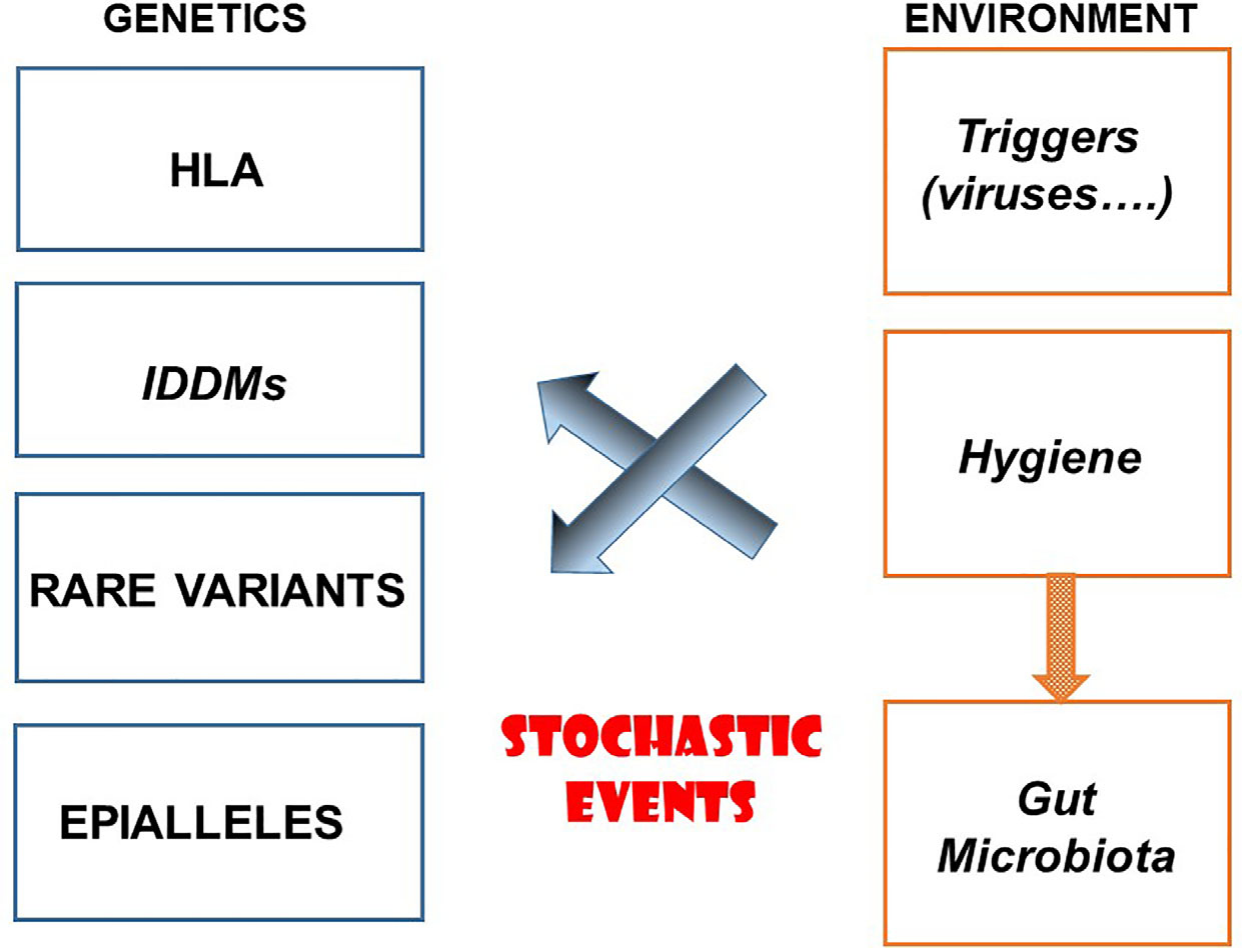
Etiological Factors for Autoimmune Diseases.

Autoimmune diseases are multifactorial and polygenic. The predisposing factors for autoimmune diseases are both genetic and environmental (**Figure 1**). Among genetic factors major histocompatibility genes (HLAs) play a major role, variable depending on the disease yet sometimes highly significant as in the case of type 1 diabetes, ankylosing spondylitis and narcolepsy. The role of a very large number of chromosomal regions identified by GWAS is certainly important, although the multitude of regions in question and the very low risk factor associated with each of them makes their priority ranking uncertain. Other genes could be involved in particular rare variants. One must also mention the potential role of epialleles that control epigenetic modifications participating to certain autoimmune diseases. Concerning the environment, a distinction must be made between factors which contribute to the onset of autoimmune diseases and those which prevent them. One must cite the hypothesis, still not proven in humans, according to which viruses could contribute to the triggering of autoimmune diseases such as type 1 diabetes and multiple sclerosis, secondarily to inflammation of the target organ by a local viral infection. The role of the gut microbiota is also relevant, although much remains to be done to demonstrate causality. To all this, we must add the possible implication of stochastic events, the existence of which is well proven in cancer (somatic mutations), still unknown for autoimmune diseases.

At this point it is important to clarify what is intended by “hygiene” when referring to the hygiene hypothesis. When speaking about hygiene, one normally thinks of classic hygiene as ensured by hand washing or social distancing rules that have reappeared in the foreground during the recent SARS-CoV-2 pandemics. The hygiene hypothesis we are discussing here is something else. It is about the environmental infectious burden which relies more on the socio-economic context specific to each industrialized country, each region or the family social context than to personal hygiene. Most of the factors that contribute to the hygiene hypothesis are collective and not individual. This infectious burden depends, to a large extent, on the quality of the drinking water, respect for the cold chain, the extensive use of antibiotics but also the generalization of vaccines. A development, obviously favorable because it prevents the occurrence of serious infectious diseases, but once again, independently of personal hygiene. The possible solutions to reduce the frequency of allergic and autoimmune diseases will obviously not come from the reintroduction of certain infections but rather from the use of “substitutes” for these infections which will retain their protective benefits.

## Interest and Limitations of Epidemiology

The hygiene hypothesis in its dynamic aspect is based on the negative correlation observed between the decrease in the frequency of infectious diseases and the increase in that of allergic and autoimmune diseases. The question arises as to whether the trends reported twenty years ago persist today (3). The answer is clearly positive concerning infectious diseases which, under the effect of hygiene, vaccinations and antibiotics, continued to decrease in industrialized countries. However, many common childhood infections persist and new pandemics such as COVID 19 occur. The question is more complex concerning allergic and autoimmune diseases. Unfortunately, there is relatively little recent epidemiological data with no international databases on these diseases as they exist for the major infectious diseases. It can be noted, however, that for T1D, as well as for allergic diseases, the frequency has continued to increase in recent years (5, 6) with, for T1D, affecting very young children (7). The question is more open for other diseases such as multiple sclerosis for which it seems, at least in some countries, that a plateau has been reached.

The main aim of the epidemiological approach to the hygiene hypothesis is to show the existence of a direct relationship between the number of infections and the frequency of allergic and autoimmune diseases. Unfortunately, it is very difficult to count infections because if one usually remembers serious infections it is much more difficult to memorize common infections which, as we have seen, probably play a significant role. Like others, we ourselves have tried to study this problem in the setting of atopic dermatitis and reached the conclusion that reliable enumeration of infections is almost impossible (8). Indirect markers must be used. The most often used concerns the socio-economic environment and family composition, in particular the number of children in families (1, 2). It is very interesting to note, as has been done for decades, that allergic and autoimmune diseases are more common in high socio-economic backgrounds and in families with few children. We find this conclusion in the study of the geographical disparity of allergic and autoimmune diseases on the one hand and infectious diseases on the other (3, 9). Several hypotheses have been put forward to explain this phenomenon (3, 9). Genetic factors do not have a determining role because migrants from countries with a low incidence of allergic or autoimmune diseases to countries with a high incidence develop these diseases with the same frequency as in host countries from the first generation (10–14). It suffices that the migration takes place before the age of 5 years for allergic diseases (14) or fifteen years for multiple sclerosis (12, 13) for the increase in incidence to manifest. This last observation, which suggests that the protective effect of infections develops over a fairly long period of childhood should be taken into consideration when discussing the role of intestinal dysbiosis insofar as the composition of the gut microbiota is fixed very early in life (2 or 3 years of age) (see below). Another interpretation calls for climatic differences. This hypothesis, which could explain the role of parasitic infections that are more frequent in tropical regions, must be considered with caution when we know that the frequency of T1D and allergies is four to six times greater in Finland than in Karelia which differ for socio-economic level while the climate and genetic factors are basically the same in these two contiguous countries (15, 16). Incidentally, one should highlight that the difference of T1D incidence between Finland and Karelia does not apply to islet-cell autoantibodies suggesting that the effect of hygiene applies more to the progression than to the triggering of the autoimmune process (17). In brief, all this suggests that it is the socio-economic factors with all the consequences on health conditions which primarily explain the differences in the frequencies of allergic and autoimmune diseases in the different regions of the world.

It would, however, be interesting to find other indirect markers of infections. This was done by analyzing the prevalence of stigmata of infections by bacteria, viruses or parasites widely distributed in the population. For example, atopy has been shown to be more common in some parts of the world when the rate of seropositivity against hepatitis A virus is low (3, 18). Regarding autoimmune diseases multiple sclerosis is associated with a lower seropositivity for cytomegalovirus (CMV) (19, 20) or Helicobacter pylori (21). The same observation was made for CMV in T1D (22). Also, multiple sclerosis is associated with an abnormally low exposure to *Toxoplasma gondii* (23).

Finally, another extremely original approach results from the analysis of the repertoire of the antigen receptor of T lymphocytes (TCR) in subjects presenting allergy. It has in fact been shown that the diversity of this repertoire was restricted which was interpreted as the reflect of a lesser solicitation of the immune system by infectious agents (24).

## Causal Relationship

It is not sufficient to observe a negative correlation between the decrease in infections and the increase in allergic and autoimmune diseases to affirm a cause and effect relationship. It is difficult to prove this in humans, although there are many arguments in favor of such interpretation. The best answer will undoubtedly come from therapeutic trials in which it will be shown directly with statistically interpretable results that the suppression of certain infections increases the frequency of allergic and autoimmune diseases or conversely that the administration of certain infectious agents or parasites, needless to say preferably in the form of extracts (25, 26), prevent their occurrence. Some elements of response have been obtained for allergic diseases, in particular worsening of asthma in patients who have been subjected to antiparasitic treatments (27). We can also mention, although the data are contradictory, the improvement in atopy observed after administration of probiotics (28). Far fewer arguments exist for autoimmune diseases. At most, one can note therapeutic trials of limited size suggesting a favorable effect of the infestation of patients suffering from multiple sclerosis by a live parasite *Tricuris suis* (29, 30). The best arguments come from studying spontaneous experimental models of autoimmunity such as the non-obese diabetic (NOD) mouse and the lupus B/W mouse. It is necessary to set aside the models of induced autoimmune diseases upon administration of autoantigens. These models use adjuvants which are known to themselves induce protection from autoimmunity (31) and therefore complicate the interpretation of a potentially preventive effect by infections. A large number of infectious agents (bacteria, viruses or parasites) prevent autoimmune disease in NOD and BW mice. We refer the reader to a recent review for the NOD mouse (9). With regard to B/W mice, kidney disease and survival can be considerably improved by viral (32) or parasitic (33) infections. It is, in fact, in this model that was published the first convincing observation of the prevention of autoimmune diseases by a parasite, in this case *Plasmodium berghi* (4). Other studies have confirmed this favorable action of parasites (25, 26, 34–36).

As we will see below, the use of these models, in addition to providing the necessary proof of concept for the hygiene hypothesis, have shed light on the underlying mechanisms.

To conclude this data clearly shows that numerous pathogenic infectious agents protect from autoimmune diseases independently of any relationship with the gut microbiota.

## Hygiene Hypothesis and Evolution

The epidemiological observations on which the hygiene hypothesis is based date back some fifty years. It is obvious that the increase in the frequency of allergic and autoimmune diseases does not have a genetic basis within populations in which changes in the frequency of these diseases have been observed, except in the case of the migrants mentioned above. These phenotypes reflect an adaptation of the organism, more particularly of the immune system, to the environment and more specifically to the infectious environment. It is, however, interesting to note that this deviance implies that the organism has adapted to changes in the environment by creating a phenotype that had not been the object of natural selection during evolution. Other examples of diseases come under the same commentary such as obesity and type 2 diabetes which are linked to overeating in individuals who have been selected to develop energy storage mechanisms (37).

On the other hand, we can ask the question of an interaction between the occurrence of infectious diseases and inflammatory diseases during evolution. This possibility has been considered in particular by L. Quintana-Murci (38, 39). This author highlighted the delicate situation in which the immune system found itself between establishing a strong inflammatory response to fight against pathogens in an environment with a high pathogen load while avoiding the harmful consequences of acute and chronic inflammation, which could lead to inflammatory and/autoimmune diseases (38). Interestingly, Genome Wide Association Studies (GWAS) studies have shown a community of single nucleotide polymorphisms (SNPs) associated with a strong anti-infective immune response and those associated with a predisposition to inflammatory and autoimmune diseases (40–42).

We can also wonder if the composition of the intestinal microbiota which, as we will discuss below, contributes to the hygiene hypothesis is not also subject to evolution, more precisely to a co-evolution of the immune system and commensal bacteria. One can imagine that the bacteria that resisted evolution did so because they had no pathogenicity and did not endanger people’s lives and could even have a favorable influence. A kind of immune tolerance has thus been created, the consequences of which are difficult to perceive and especially the relationship with the role of the microbiota in the hygiene hypothesis.

Asking this question leads us to evoke the selective pressure that has weighed on the human species since the appearance of *Homo-sapiens* (38, 39). In recent years, population genetics work has provided major information thanks to advances in genomics. First, it should be noted that modern *Homo-sapiens* arose from crosses between African humans and Neanderthals. Neanderthals contributed little (about 2%) to the *Homo-sapiens* genome. Nevertheless, it has been repeatedly shown that some of the haplotypes which persist in *Homo-sapiens* and which originate from Neanderthals include genes important for immune responses. It has recently been shown that certain genes predisposing to COVID 19 are derived from Neanderthals (43). Subsequently, over the past 100,000 years, genes involved in immune reactions have evolved through a process of natural selection, positive or negative (purifying). Evolution has taken place under the selective pressure of environmental conditions, first the shift from humans hunters/collectors to humans cultivators, then the migration of humans from Africa to Asia and then to Europe. Numerous studies carried out on this subject, in particular by L. Quintana-Murci, have made it possible to identify the genes in question (38, 39). These are essentially genes controlling innate immunity. We note more particularly the presence of genes encoding toll-like receptors (TLRs). It thus appears, which was intuitive, that the evolution gave rise to an improvement of the immune responses against infectious agents under their pressure. The case of epidemics illustrates this point. Thus, subjects with a mutation in the NOD-2 gene, associated with inflammatory bowel diseases, seem to be over-represented in populations that have been severely affected by plague epidemics (44).

How, then, can these observations be linked to the hygiene hypothesis? It is important to note that the changes in the genes of immune responses during evolution have been made at a particularly rapid rate. These changes did not span hundreds of thousands of years as is the case for other genes but often only a few thousand years, and sometimes even less in major epidemics or extreme environmental situations. In contrast, the hygiene hypothesis is based on adaptation rather than selection. We thus find ourselves in a situation where the time scale associated with the selective pressure which influenced natural selection intersects with that of the hygiene hypothesis which only applies to the last 50 years. Going further, one can wonder whether the reduction in the infectious burden, on which the hygiene hypothesis is based, will not influence the evolution of the genes controlling the immune responses. Conversely, even if these diseases were serious enough to reduce the number of offsprings in affected subjects, the genes which control them are so numerous and interactive that it is difficult to see how they could influence natural selection, especially since they are rarely deleterious mutations but more often polymorphisms whose isolated presence in healthy subjects has no consequence.

## Intestinal Dysbiosis, Cause, Consequence, or Modulation

The emergence of metagenomics, which has made it possible over the past fifteen years to characterize the composition of the intestinal microbiota, has opened a new page in the hygiene hypothesis. Several arguments, suggest that a decrease in the diversity of the intestinal microbiota could contribute to the occurrence of autoimmune diseases as well as many other pathologies including allergic diseases, type 2 diabetes and obesity.

The mainstay is the existence of a dysbiosis, that is to say an imbalance of the commensal bacteria that make up the intestinal microbiota in the diseases in question. One observes, indeed, in these different diseases a reduction in the diversity of the microbiota, more particularly in certain species, with often a decrease in lactobacilli (45), in particular in type 1 diabetes (9), multiple sclerosis (46–48) and systemic lupus erythematosus (49–51).

At the same time, destruction of the gut microbiota by administration of broad-spectrum oral antibiotics to mothers and newborns has been shown to increase the frequency of T1D in NOD mice (52) and experimental asthma (53).

The link between these observations on the hygiene hypothesis quickly became apparent. We know, in fact, that the composition of the intestinal microbiota is different depending on the level of hygiene. This has been demonstrated in particular by comparing the microbiota of subjects living in Italy or Burkina Faso (54), an observation which is, however, not conclusive because many other elements differ between such countries, in particular diet which influences the composition of the microbiota. A more direct argument is the observation that pigs reared in a clean facility have a different microbiota than those reared in a conventional barn (55).

These observations aroused great enthusiasm. Numerous studies have attempted to characterize commensal bacteria which could be responsible for regulating autoimmunity and whose absence or decrease could contribute to the occurrence of autoimmune diseases. It must be recognized, however, that so far, few conclusive results have been reported.

To these fairly convincing arguments it is necessary to mention other elements which incite more reticence. The main question is that of the causal relationship between dysbiosis and the occurrence of the diseases under consideration. Does said dysbiosis play a role in triggering the disease or in its progression or is it the consequence of the disease. To answer this question, we should not be content to study the composition of the microbiota at the time when the disease is already declared. The microbiota should be studied before the onset of the disease. In fact, this has so far only been done extensively in T1D, where cohorts of subjects with a high inheritance of diabetes have been followed from birth. Dysbiosis has been observed at the time of disease onset (56). However, if we carefully examine the timing of the onset of dysbiosis, we find that it takes place after the development of the autoimmune response against the beta-cells of the islets of Langerhans, suggesting either that it is secondary to the inflammation associated with the onset of diabetes or it contributes to the transformation of respectful insulitis into malignant insulitis which marks the onset of clinical diabetes.

The conclusions from the use of probiotics remain uncertain. The probiotics used were not really calibrated and the number of bacteria administered was very low compared to the number of intestinal bacteria, posing the problem of their mode of action: modification of the composition of the microbiota, but then for how long, pharmacological effect or other mechanisms.

In brief, there are interesting arguments to suggest the role of dysbiosis in the occurrence of autoimmune diseases, but they are fragile. In any case, data suggests in view of the results obtained in animal models that pathogens also play an important role, in particular agents that have no relation to the intestine such as mycobacteria.

## Multiple and Complex Underlying Mechanisms

Many publications have been devoted to the mechanisms underlying the hygiene hypothesis namely, how to explain that infections can reduce the frequency of allergic or autoimmune diseases. It appears very clearly that no univocal explanation can be presented for all the protective effects of infections. Several major mechanisms appear to operate. The problem is complicated by the fact that the mechanisms can be different depending on the infection. We will briefly discuss the main data available by referring the reader who would like more details and a more documented bibliography to a general review recently published (9).

It has been known for many decades that concomitant immune responses compete, a phenomenon termed antigenic competition which could well be applied to the hygiene hypothesis by supposing that very strong immune responses against infectious agents could compete with immune responses directed against weak antigens such as allergens or autoantigens due to increased consumption of homeostatic factors. In fact, this mechanism has been studied very little in the case of the hygiene hypothesis. This may be explained by the poor knowledge that we still have today on the molecular basis for antigenic competition, even if converging data seem to give a major role to homeostatic factors, in particular interleukin (IL)-2, IL-7 and IL-15. In any event, as attractive as it is, this hypothesis remains very poorly documented.

Before attempting to present a unitary hypothesis, it is important to mention that certain mechanisms appear to be relatively specific to certain infectious agents. Thus, the protective effect of live mycobacteria or Freund’s complete adjuvant involves CD4+CD25+FOXP3+ regulatory T lymphocytes like other infectious agents but also, more unexpectedly, natural killer or NK cells (57). Lipopolysaccharide (LPS) contained in *Escherichia coli* similarly stimulates regulatory T lymphocytes and it also stimulates a particular subset of IL-10-producing B lymphocytes which play an important immunoregulatory role (58).

The commensal bacteria of the intestinal microbiota also have a protective effect against autoimmune diseases. This has been shown globally with probiotics (9) but also with well-identified commensal bacteria (lactobacilli). In this case too, various mechanisms are involved. As far as probiotics are concerned, a predominant role has been attributed to IL-10. In other situations, such as Clostridium, a major role has been ascribed to CD4+CD25+ FOXP3+ regulatory T cells (59). It should also be noted that many infectious agents modulating autoimmune responses involve the immunoregulatory cytokine TGF beta. This is the case of a gram-positive bacterial extract which protects the NOD mouse from diabetes, an effect which is reversed by the administration of antibodies neutralizing TGF beta but not IL-10 (60). Moreover, a key role of TGF beta has also been shown in the protective mechanisms mediated by various parasites with the exception of schistosomes which appear to act through IL-10 production (61). A role for interferon gamma (IFN*γ*) has been proposed for both allergy (62) and autoimmunity (63). In brief, multiple mechanisms are involved that mostly rely on immunoregulatory circuits.

At the molecular level, many arguments suggest that both pathogens, bacteria or viruses and also parasites as commensal bacteria exert their protective effects by primarily involving their molecular interactions with TLR receptors. These different infectious agents indeed contain TLR ligands, both pathogens and commensals. Above all, the systemic administration of chemically characterized ligands of the various TLRs reproduces the protective action of the infectious agents mentioned above (64). It should also be noted that it is not necessary for the infectious agents in question to be alive to prevent the onset of autoimmune diseases: they can be substituted with bacterial (60) or parasitic extracts which have the same effect (25, 26). The different TLR ligands could have distinct mechanisms of action depending on the specific receptor involved. For example, TLR4 ligands appear to act through FOXP3+ regulatory T cells, while TLR3 ligands involve invariant NKT cells (64).

It is now well known that the desensitization of macrophages to the pro-inflammatory effect of LPS, also called “endotoxin tolerance”, is mediated by a joint action through TLR4 and TLR2 receptors and their common signaling pathways (65). Another example of desensitization is the prevention of type 1 diabetes in NOD mice by TLR2 ligands, called “TLR2 tolerance” (66). In an adoptive transfer model, it was shown that the repeated treatment with the TLR2 agonist Pam3CSK4 of NOD mice receiving diabetogenic T lymphocytes inhibited the development of the disease (66). This same TLR2 desensitization could also be involved in the prevention of experimental allergic encephalomyelitis (EAE) (67). Administration of low doses of two different TLR2 ligands, Pam2CSK4 or Lipid 654 (L654), to naive recipients of encephalitogenic (EAE-inducing) T cells decreased the level of TLR2 signaling at the same time as it attenuated EAE (67, 68). Interestingly, L654 is a TLR2 ligand derived from a commensal of the microbiota which is present in healthy human serum, but whose concentration is significantly reduced in the serum of patients with multiple sclerosis (67, 68). In another mouse model of EAE, repeated administration of a synthetic TLR7 ligand has been reported to significantly decrease the severity of disease as well as the expression of chemokines in the target organ (69). Finally, it is interesting to note that NOD mice genetically deficient in TLR4 and MyD88 show an acceleration in the severity of diabetes and experimental asthma respectively, again suggesting a protective role for TLR signaling (64, 70).

In humans, the role of desensitization by TLRs has been demonstrated in an elegant work by the group of B. Lambrecht concerning children raised on dairy farms (an environment rich in LPS) who present a low incidence of allergy (62, 71). Epithelial cells in the lungs have shown reduced production of cytokines that normally activate dendritic cells to induce TH2-type lymphocyte responses. The TLR4 desensitization induced by LPS which could be responsible for this effect targets the pulmonary epithelium (71).

In conclusion, although very interesting, this data do not suffice to give a complete picture of the mechanisms underlying the hygiene hypothesis. It is therefore necessary to consider the overall response to pathogens or commensals as the result of the integration of positive and negative signals delivered *via* the TLRs. This concept paves the way for a TLR targeted immunopharmacology.

## Conclusions

The hygiene hypothesis, misnamed as it is, teaches us a lot about immunity, immunopathology, epidemiology and evolution. It indicates how flexible the immune system is, under constant control by immunoregulation under the influence of the environment. It sheds light on the mechanisms underlying many immune related diseases, in particular autoimmune diseases but also probably many other diseases which involve an uncontrolled differentiation of lymphocytes, whether they are allergic diseases or certain malignant lymphoproliferative disorders (72). It opens new perspectives on the etiological factors of autoimmune diseases by distinguishing those that could trigger or on the contrary protect (**Figure 1**). Finally, when it comes to evolution, it provides a particularly bright illustration of the relationships that may exist between natural selection and adaptation under the control of infectious agents.

## Author Contributions

The author confirms being the sole contributor of this work and has approved it for publication.

## Funding

The laboratory of the author was supported by an advanced grant from the European Research Council (ERC, Hygiene N°: 250290).

## Conflict of Interest

The author declares that the research was conducted in the absence of any commercial or financial relationships that could be construed as a potential conflict of interest.
